# Psychophysics and computational modeling of feature-continuous motion perception

**DOI:** 10.1167/jov.22.11.16

**Published:** 2022-10-28

**Authors:** Felix M. Töpfer, Riccardo Barbieri, Charlie M. Sexton, Xinhao Wang, Joram Soch, Carsten Bogler, John-Dylan Haynes

**Affiliations:** 1 Charité – Universitätsmedizin Berlin, corporate member of Freie Universität Berlin, Humboldt-Universität zu Berlin and Berlin Institute of Health (BIH), Bernstein Center for Computational Neuroscience, Berlin Center for Advanced Neuroimaging, and Department of Neurology, Berlin, Germany; 2 Melbourne School of Psychological Sciences, The University of Melbourne Parkville, Melbourne, Australia; 3 Humboldt-Universität zu Berlin, Berlin School of Mind and Brain and Institute of Psychology, Berlin, Germany; 4 Research Group Cognitive Geriatric Psychiatry, German Center for Neurodegenerative Diseases, Göttingen, Germany; 5 Technische Universität Dresden, SFB 940 and Cognitive Control, 01069 Dresden, Germany

**Keywords:** continuous judgment, motion perception, random dot kinematogram (RDK), bias, von Mises mixture model (vMMM)

## Abstract

Sensory decision-making is frequently studied using categorical tasks, even though the feature space of most stimuli is continuous. Recently, it has become more common to measure feature perception in a gradual fashion, say when studying motion perception across the full space of directions. However, continuous reports can be contaminated by perceptual or motor biases. Here, we examined such biases on perceptual reports by comparing two response methods. With the first method, participants reported motion direction in a motor reference frame by moving a trackball. With the second method, participants used a perceptual frame of reference with a perceptual comparison stimulus. We tested biases using three different versions of random dot kinematograms. We found strong and systematic biases in responses when reporting the direction in a motor frame of reference. For the perceptual frame of reference, these systematic biases were not evident. Independent of the response method, we also detected a systematic misperception where subjects sometimes confuse the physical stimulus direction with its opposite direction. This was confirmed using a von Mises mixture model that estimated the contribution of veridical perception, misperception, and guessing. Importantly, the more sensitive perceptual reporting method revealed that, with increasing levels of sensory evidence, perceptual performance increases not only in the form of higher detection probability, but under certain conditions also in the form of increased precision.

## Introduction

Studies on visual motion perception and perceptual decision-making have frequently focused on binary or multiple-alternative choice tasks, which require participants to choose among a fixed set of stimulus categories (Ball & Sekuler, 1979; Braddick, 1973; Hebart, Donner, & Haynes, 2012; Palmer, Huk, & Shadlen, 2005; Sekuler & Ganz, 1963; Urai, Braun, & Donner, 2017; Watamaniuk & Sekuler, 1992). However, human motion perception is inherently continuous across the space of directions (Levinson & Sekuler, 1976). Therefore, when probing a continuously distributed stimulus feature such as motion, a continuous report task should provide more detailed information than a categorical one. Indeed, it allows measuring the full distribution of an observer's response deviations in a graded fashion (e.g., Prinzmetal, Nwachuku, Bodanski, Blumenfeld, & Shimizu, 1997), which allows to assess precision and to identify systematic biases.

An important question when studying the perception of continuous features is what the best method is for obtaining the graded perceptual reports. It would be unfortunate to lose the benefits of continuous reports by the potential distortions introduced by the mode of reporting. Several approaches could be used in principle. First, one could simply ask a participant to indicate the perceived direction of a motion stimulus with a movement, say using a trackball. Such a purely motor-based response (Menozzi, Huang, & Abt, 2016) involves a coordinate transform from a perceptual to a motor coordinate system, which might limit precision. Importantly, the directional limb movement that is necessary to move an analogue device, like a trackball, mouse, or joystick, can introduce additional motor variability or biases (Engen, 1971; Green & Swets, 1966; Menozzi et al., 2016; Smith, 2016). However, it has the advantage that it can be performed without presenting competing visual stimuli, thus decreasing the potential effects of interference of attention on perceptual performance. A second approach is to avoid coordinate transforms between modalities altogether and ask participants to report the perceived direction using a visual comparison stimulus (as discussed elsewhere in this article). This approach would introduce additional perceptual load and potential interference within the visual system, but it would avoid the problem of transforming between visual and motor coordinate frames, and averts differential motor movements.

In most studies that use continuous reports, the tasks mix the two approaches by combining a direction-dependent movement with a visual comparison stimulus. For example, when subjects or animals are asked to respond by moving a visible pointer on the screen using a joystick, mouse, or trackball in the direction of choice (e.g., Nichols & Newsome, 2002). The relative contribution of perceptual or ergonomic motor components to continuous behavioral judgments thus remains to be clarified. For this reason, here we used two more pure forms of reporting: a motor reference frame without visual feedback and a perceptual reference frame where the response does not require a differential movement.

We used motion direction as a continuous feature because, other than orientation, it spans a full space of 360°, and other than color, it is not perceived to fall into distinct categories, thus introducing additional biases (Bae, Olkkonen, Allred, Wilson, & Flombaum, 2014). Specifically, we used random dot kinematograms (RDKs; Newsome & Pare, 1988), which have been used extensively to investigate global motion perception, both in humans and in animals (Britten, Shadlen, Newsome, & Movshon, 1992; Watamaniuk & Sekuler, 1992). Several studies have assessed the differences between the most common variants of motion displays with inconclusive results (Pilly & Seitz, 2009; Scase, Braddick, & Raymond, 1996; Schütz, Braun, Movshon, & Gegenfurtner, 2010; Snowden & Braddick, 1989; Watamaniuk, Sekuler, & Williams, 1989). The application of a continuous task design for motion stimuli allows to model behavioral responses and to assess the underlying parameters (Zokaei, Gorgoraptis, Bahrami, Bays, & Husain, 2011) that might help to differentiate RDKs more conclusively.

We tested six groups of participants in a decision-making experiment where the direction of motion changed from trial to trial, spanning the full range of motion directions (between 0° and 360°). Each group was assigned in a between-subject design to a combination of two experimental factors: one of three different RDK types (transparent motion [TM], Brownian motion [BM], and limited-lifetime white noise motion [WM]; for details see the Materials and Methods section, Visual stimuli), and one of two alternative response methods. The first required participants to move a trackball in the direction of choice without a corresponding pointer on the screen—thus, responding in a motor frame of reference. The other used a visual comparison stimulus. Specifically, participants had to press a single button when a rotating bar pointed in the perceived direction of the target—thus, responding in a perceptual frame of reference without a direction-specific movement.

We found that responses in the motor frame were biased strongly in the direction of the vertical axis of the trackball and possessed a consistent rotational bias in reference to the presented stimulus directions. Responses obtained with a perceptual frame didn't have these biases and were more precise (as discussed elsewhere in this article). Unexpectedly, and independent of the response method, we found evidence for a systematic misperception of motion direction where subjects occasionally perceived the motion as in the exact opposite direction, a phenomenon we refer to as “ROOD” (for report of opposite direction) (Bae & Luck, 2018; Barbieri, Töpfer, Soch, Bogler, & Haynes, 2018). Note that one would be unable to distinguish between such a systematic misperception in the opposite direction and a random guess in categorical tasks. This finding was corroborated with a five-parameter von Mises mixture model (vMMM) that assessed the contribution of three different processes to the response distribution: detections, pure guessing and systematic misperceptions. Using a perceptual frame report, which is more sensitive, the estimated parameters suggest an increase in detection probability combined with an increase in detection precision for BM and WM. Detection probability increases for TM as well, although there is no evidence that detection precision is dependent on coherence level. Together, this finding suggests that there are different trade-offs between detection frequency and detection precision between the tested RDKs.

## Materials and methods

### Participants and group assignment

We recruited participants from students and other people, some of whom had participated in previous experiments in our laboratory. All of the participants gave written informed consent and were paid 7€/h. The research protocol was conducted in accordance with the Declaration of Helsinki and it was approved by the ethics committee of the Humboldt University of Berlin. Only healthy right-handed subjects with no history of neurological or psychiatric diseases were selected. Participants were pseudo-randomly assigned to a specific combination of RDK, TM, BM, and WM and response type (motor frame of reference, trackball; perceptual frame of reference, rotating bar), maintaining the balance between males and females across groups. This yielded six experimental groups: TM–trackball, TM–rotating bar, BM–trackball, BM–rotating bar, WM–trackball, and WM–rotating bar. Based on the sample size of a similar experiment (Pilly & Seitz, 2009) and previous pilots performed in our laboratory, we enrolled 12 participants for each condition. During the experiment, one subject was excluded who found the paradigm too difficult and started to respond randomly after the seventh run (condition: BM–rotating bar). A new subject replaced this subject. We finally analyzed the data of 72 subjects (34 male, 38 female; age range 18–35 years; mean age, 26.18 ± 4.25 years).

### Experimental setup

The experiment took place in a dimly illuminated room (luminance on the white wall behind the screen of 0.0998 cd/m^2^). Participants were sitting 60 cm away from a 19" DELL LCD monitor, with a 60 Hz refresh rate and resolution of 1,280 × 1,024. The distance from the screen was fixed by placing their head on a chinrest with a head bar. The stimuli were generated and presented using MATLAB 2016a (The MathWorks Inc., Natick, MA) and the extensions Psychtoolbox 3 (Kleiner et al., 2007) and CircStat-Toolbox (Berens, 2009). Participants assigned to the trackball groups laid their right hand on a Kensington 64327EU 40-mm trackball fixed on the right side of a desk with a Velcro strip. The trackball fixation allowed to constrain the arm position and minimize random rotational biases. Participants assigned to the rotating bar groups placed their right hand on a standard computer keyboard positioned in front of them.

### Visual stimuli

**Figure 1.**
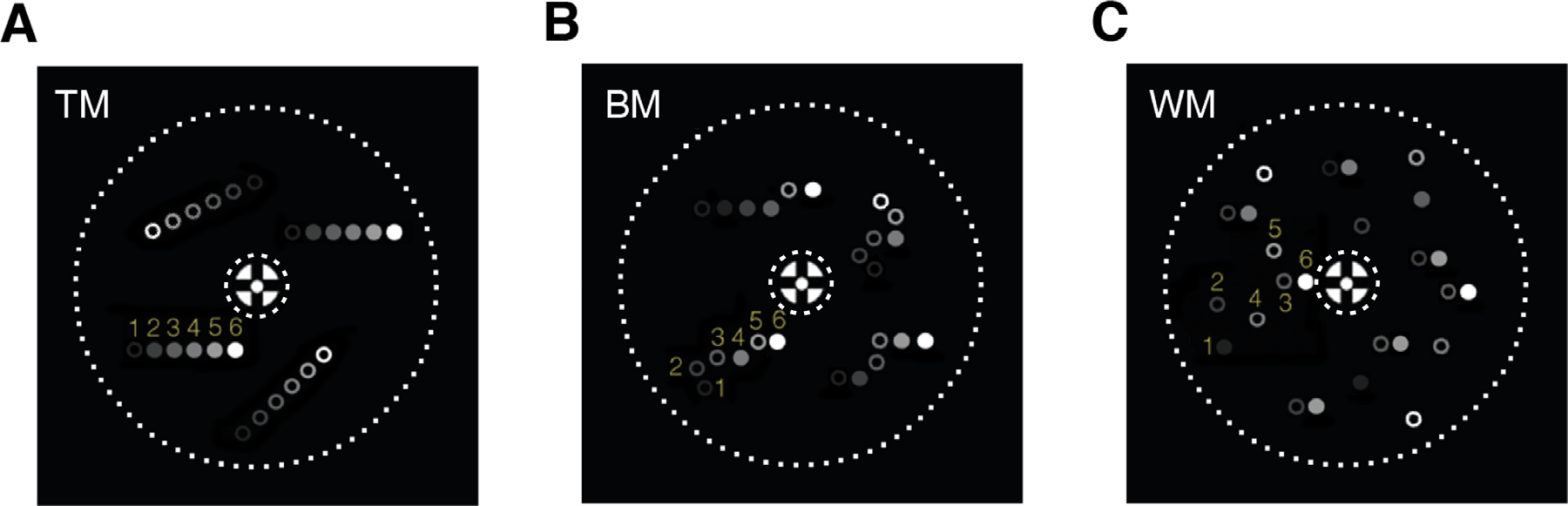
Schematic depiction of six frames of each RDK implementation (A– C) with signal dots moving rightward at 50% coherence. Filled dots represent signal, and empty dots represent noise. The brightest dots represent those displayed in the sixth (final) frame, and the decreasing contrast levels mark the five previous frames. This strategy allows seeing the geometry of potential motion traces that could occur owing to temporal integration in the visual system. Note that the fading of the dots in this picture does not represent the alphablending applied to the dots on the inner and outer boarder of the stimulus during the experiment, but instead represents consecutive presentation frames. For each motion type, one motion trace is indicated by the numbers from 1 to 6. The dotted circle represents the outer and inner boundary of the stimulus. (A) In TM stimuli, signal and noise dots move in their assigned directions—signal or random directions, respectively—for six consecutive frames. This continuity leads to the perception that the stimuli consist of a plate of signal dots and another transparently overlaid plate of noise dots. (B) In BM stimuli, each dot is assigned to move in the signal direction or a random direction newly every single frame, resulting in the characteristic random walk movement. (C) In WM stimuli, three completely uncorrelated sets of dots are plotted sequentially. The consecutive positions of the dots of a single set are presented after the other sets of dots have been plotted. In the figure, the motion step happens between the third and the sixth frames of the numbered exemplary trace (for details see the Materials and Methods section, Visual stimuli).

The RDKs consisted of 275 white dots moving in a black circular aperture centered on the screen (schematically shown in Figure 1) with an inner diameter of 2.5 degrees of visual angle (dva) and an outer diameter of 15 dva. The aperture was overlaid with an invisible mesh containing a circular logarithmic kernel overlaying the inner and the outer boarder of the aperture. This mesh, effectively looking like an invisible donut, held the transparency values of the dots at certain positions in the aperture. Specifically, when a dot approached the inner border, it was successively blended out until it visually fully disappeared. Invisibly, it continued its path through the inner diameter and was slowly faded in once it crossed the border of the inner diameter. Instead, when a dot approached the outer border, it was successively faded out as well, but was wrapped around to the other side of the aperture where it started to fade in again. This alpha blending, at the inner and the outer border of the aperture, was applied to reduce possible end-stopping effects.

The dot size and the dot step size were set to 0.1 dva (Braddick, 1973). The dots' speed of motion was 6°/s (Geisler, 1999; van de Grind, van Doorn, & Koenderink, 1983), and the dot density was 1.6 dots/dva^2^ (Downing & Movshon, 1989). A white bulls eye fixation target (Thaler, Schütz, Goodale, & Gegenfurtner, 2013) was placed in the central aperture that spanned 1 dva. The mean luminance of the white center of the bulls eye was 17.5 cd/m^2^. The mean luminance measured of the black background was 0.206 cd/m^2^.

For the randomization of continuous directions, the motion directions of the stimuli were separated into eight directional randomization bins. Each bin divided the stimulus into equal sectors of 45°. The borders between neighboring bins were set at 22.5°, 67.5°, 112.5°, 157.5°, 202.5°, 247.5°, 292.5°, and 337.5°. Within each bin, single directions were drawn randomly from a uniform distribution. In this way, we made sure that the motion direction would have varied continuously across trials while respecting some experimental constraints: 1) Each subject saw an equal number of trials in each directional bin, and 2) in consecutive trials, the direction was never sampled more than two times from the same directional bin, and it never appeared the same coherence more than three times in a row.

In RDKs, signal dots move in the target direction, and noise dots move in any other random direction following a stimulus-specific pattern (as discussed elsewhere in this article). The coherence level defines the relative proportion of signal and noise dots. The general features of stimuli (contrast, speed, density, dot size) were fixed across groups. Each group was presented with one of the three different RDK types (Pilly & Seitz, 2009; Scase et al., 1996).

(1) In the *TM* *stimulus*, each dot was either assigned a signal dot or a noise dot at the beginning of a 100-ms cycle. The dots kept the same identity and direction for each consecutive step describing a straight trajectory for 100 ms (six frames) before the cycle resets (Figure 1A). The limited lifetime made it difficult for the subject to track individual dots and infer the overall motion direction (compare with Scase et al., 1996; noise type: “random direction,” signal selection: “same”).
(2) In the *BM* *stimulus*, each dot was assigned to be either signal or noise, similarly to the TM (Pilly & Seitz, 2009). However, the dot identities in BM were shuffled in every frame. In this way, the overall proportion of signal and noise dots remained constant, and the perceptual outcome was a characteristic random walk (Figure 1B). Owing to this frame-by-frame change of identity, a subject would not have been able to infer the global motion direction by tracking single dots even across short time periods. Furthermore, the trajectories of the dots were monitored across frames, and each dot that moved more than 34° within 100 ms was erased (Watamaniuk et al., 1989). The lifetime of a dot could span from a minimum of 100 ms to a maximum of 200 ms, with lower coherence levels having a longer average lifetime than higher coherence levels. Note that, at 100% coherence, all of the dots in the BM RDK constituted the signal and moved in the same direction (compare with Pilly & Seitz, 2009; BM). In this condition, the stimulus was the same as the one produced by the TM RDK.
(3) The *WM* *stimulus* differed more substantially from the TM and BM (Pilly & Seitz, 2009). To create a WM stimulus, three independent sets of dots were generated. Each set contained the same number of dots and the same proportion of signal and noise dots, depending on the coherence level. During the presentation, in the first frame, the first set of dots was presented. In the next two frames, the two remaining sets were plotted sequentially. This sequential plotting of the three utterly uncorrelated sets of dots leads to a characteristic percept that resembles a snowstorm, because it seems as if the dots are jumping around independently on the screen (Figure 1C). Note that, in this stimulus, the apparent motion is induced across gaps of three frames. After the second presentation of each set of dots, the dots' identities (signal or noise) were reshuffled. Therefore, the lifetime of a single dot was only 67 ms (four frames), conveying a single step of motion, before it was assigned to a new identity (compare to Pilly & Seitz, 2009; Limited Lifetime, LL). The implementations of all stimuli were based on code presented in Urai et al. (2017).

### Response methods

#### Trackball groups (motor frame of reference)

**Figure 2.**
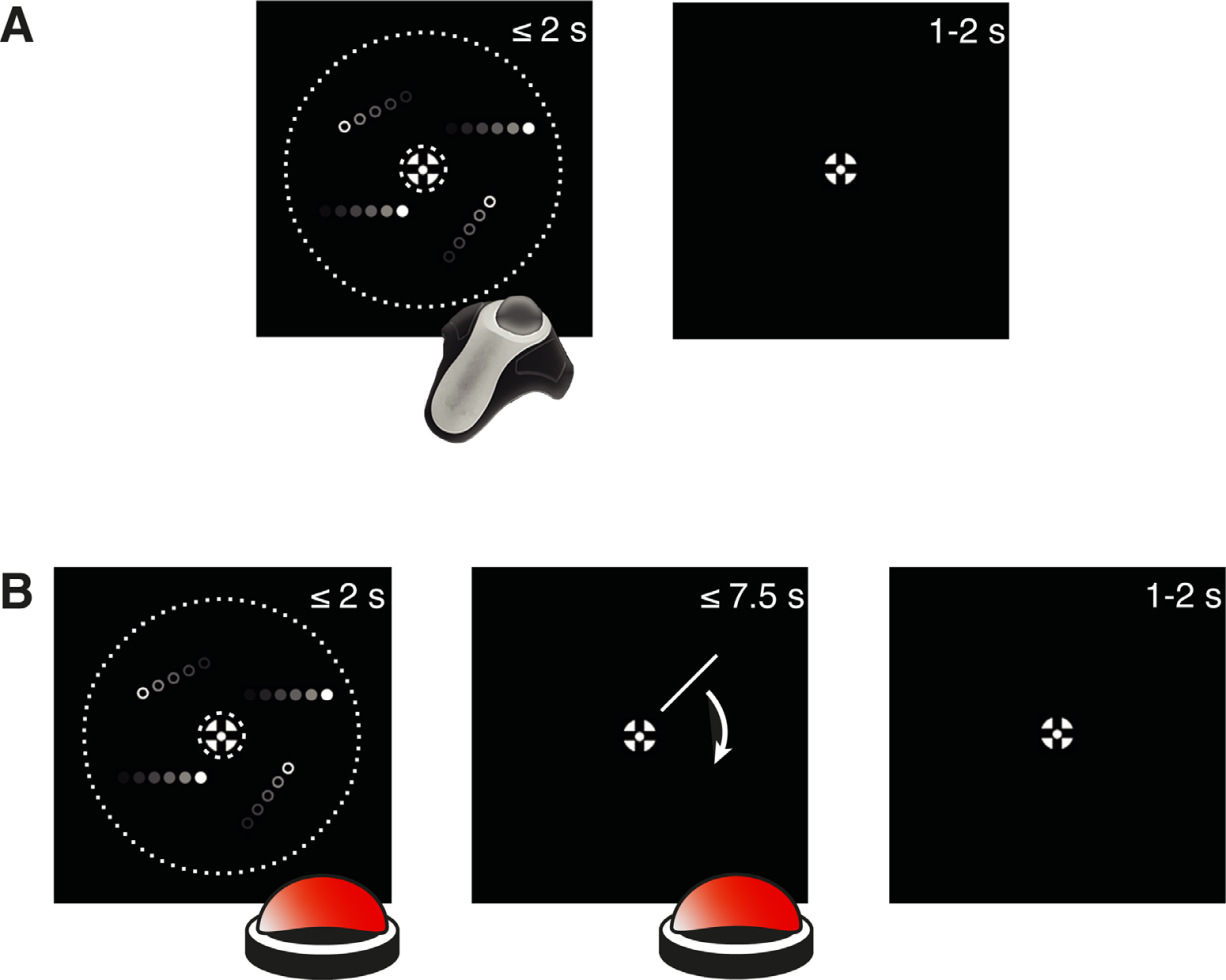
Illustration of one experimental trial separately for each of the two response methods. (A) Experimental trial for responses recorded with the trackball. After the onset of the RDK, subjects were asked to respond during the stimulation (maximum of 2 seconds) by rotating the trackball in the direction of choice. (B) Experimental trial for responses recorded with the rotating bar. After the onset of the RDK, subjects were asked to press a button during the stimulation to indicate that they had made their decision (maximum of 2 seconds). Consecutively, a bar appeared that could rotate clockwise (presented in the figure) or counterclockwise around the fixation. When the bar pointed in the direction of choice, subjects were asked to press the button a second time. The images are not to scale and do not truly reflect the features of the stimuli or the devices used in the experiment.

Three groups of participants (one with each motion type) reported the perceived motion direction using the 360° directions of a trackball (Figure 2A). Participants were instructed to place the index and middle finger on the device's ball and move it in the desired direction as fast and accurately as possible to make a report. There was no visual feedback as to the direction of movement. The rotation of the trackball moved an invisible cursor on the screen. On each trial, the starting position of the invisible cursor was reset to the center of the fixation appearing on the screen. Responses were recorded, and the stimulus terminated, at the moment the distance of the cursor from the center of the fixation exceeded 1 dva. This criterion was necessary to impose a threshold so that the report was independent of small and unspecific finger movements of the subject during the stimulation period. The recorded response was then calculated as the central angle between 0° and the position where the invisible cursor hit the invisible circle with a radius of 1 dva.

#### Rotating bar groups (perceptual frame of reference)

Three other groups of subjects (one for each motion type) were asked to report the perceived motion direction using a sequence of two button presses on the spacebar of the keyboard with their right index finger: The first time they pressed the button to indicate that they had made up their mind. Immediately afterward, a bar that was 7.5 dva in length appeared on the screen and rotated either clockwise or counter-clockwise around the central fixation at a speed of 0.2 cycles per second. The rotation direction (clockwise or counterclockwise) was assigned randomly in each trial and counterbalanced across each run to avoid systematic biases. Participants were asked to press the spacebar a second time when the rotating bar matched the direction of motion they perceived. Importantly, the bar was pointing from the center of the aperture to the outer border of the stimulus (like the arm of a clock), starting from a random direction in every trial. The rotation proceeded until the subject placed a decision, or it kept rotating for a maximum of 1.5 cycles (Figure 2B).

### Experimental task and testing procedure

Subjects were cued to the start of a new run by the presentation of a fixation cross for 1 second followed by a bulls eye for one additional second. Subsequently, an RDK was presented for a maximum of 2 seconds, during which the participants had to decide on the net direction of motion. Subjects were allowed to respond with the assigned method in the entire interval from the onset of the stimulus until its offset (for details, see the Materials and Methods section, Response methods). We asked them to report the perceived motion direction as fast and accurately as possible. The response was followed by a uniform random intertrial interval of 1 or 2 seconds, after which a new trial began (Figure 2). We asked the participants to maintain fixation for the whole duration of the experiment. This was encouraged by presenting a fixation bulls eye in the center of the screen during every task trial.

Every experiment started with a practice run, where subjects had to perform 40 trials at full coherence (100%). The practice run was designed to test whether participants were able to correctly identify the stimulus and to effectively use the assigned response method (trackball or rotating bar).

After the practice runs, participants were tested on five levels of coherence (0%, 12.5%, 25%, 50%, and 100%) that were pseudorandomly chosen during a run with the constraint that one coherence level never appeared more than three times in a row (for details see the Materials and Methods section, Visual stimuli). Each subject performed 14 runs with 40 trials per run, yielding a total of 560 trials. To keep the motivation up, after every 10 trials of a run subjects obtained feedback on the screen. The presented accuracy was calculated as the fraction of correct responses from the presented trials of this run. For all subjects, correct responses were initially leniently defined as those with a response deviation of less than 60° (out of a maximum of 180° possible in either direction). The range of correct responses was adapted after the second and the fourth runs. Here we calculated the two standard deviations of the distribution of response deviations at 100% coherence. The obtained value was used as a new criterion for correctness in the subsequent runs, but it was only adapted if it was less than 60°. This procedure allowed us to adjust the feedback for subjects with good performance while avoiding discouraging participants with lower performance. After the fourth run, the feedback criterion remained constant throughout the last ten runs. The first four runs were training runs and they were excluded from subsequent analyses.

### Target and response distributions

It has been pointed out previously that movement can confound behavior reports with motor variability and bias (Engen, 1971; Green & Swets, 1966; Menozzi et al., 2016; Smith, 2016). We tested for the presence of biases by comparing the observed distributions of reports (*F_m_*) with the near-uniform distributions of presented stimuli (*F_n_*) for all coherence levels using a Cramér von Mises test (Anderson, 1962; Watson, 1961). This test allowed us to evaluate biases by testing for the difference between two continuous distributions (*F_n_*, *F_m_*) by calculating the integral of their squared differences.

(1) $$\omega^{2}=\int_{0}^{2\pi }{\left[F_{n}\left(x\right)-F_{m}\left(x\right)\right]}^{2}dF_{m}\left(x\right).$$

Note that, owing to the random nature of trial-wise stimulus direction selection (for details see the Materials and Methods section, Visual stimuli), there are very slight deviations from uniformity in the sample distribution of presented directions (*F_n_*). That is why the comparison is made to the actual near-uniform distribution of presented directions, not to the uniform distribution itself.

### Reaction times (RTs)

The RT was recorded for each trial. For the trackball groups, the RT was considered the duration between the stimulus onset and the moment in which the trackball rotation moved an invisible cursor 1 dva away from the center of the stimulus. This criterion was necessary to allow for small, unspecific finger movements of the subject during the stimulation period. For the participants responding with the rotating bar, the RT was defined by the time between the stimulus onset and the first button press.

### Modeling behavioral performance

In a feature-continuous study, the physical stimulus directions and the subjects' responses can be placed in all possible directions. This design allows a graded measure of performance by estimating the trial-wise response deviation Δθ obtained by subtracting θ_*s*_ (the stimulus direction) from θ_*r*_ (the reported motion direction). Modeling the distribution of response deviations can be a source of information about behavioral performance. This idea has been adopted from studies using continuous visual features in the field of working memory, where response deviations have been modeled as a mixture of true recognitions and guessing (Zhang & Luck, 2008). For reasons that will become clear elsewhere in this article, we refined a recent approach to model distributions of response deviations using a vMMM, assuming that in each trial, perceptual choices might originate in one of three (instead of just two) different processes (Bae & Luck, 2018, 2019). More specifically, we hypothesized that the distribution of response deviations consisted of three components: i) detection (trials in which the direction of motion was correctly identified with a specific precision), ii) ROOD (reports of opposite direction with some precision; as discussed elsewhere in this article), and iii) guessing (trials in which subjects were purely guessing and the choice was thus independent of the presented direction).

The parameters of these components were estimated from the distributions of response deviations by fitting a vMMM mixing three distributions: i) a von Mises distribution (vM) describing detections with mean centered around Δθ = 0°, bias μ and precision κ_1_; ii) a second vM distribution describing ROODs with mean centered around Δθ = 180°, the same bias μ and a different precision κ_2_; and iii) a continuous uniform distribution (U) between 0° and 360° describing guesses. Each component received a separate mixture coefficient *r* (with *r*_1_ + *r*_2_ + *r*_3_ = 1; *r*_1_, detection; *r*_2_, ROOD; *r*_3_, guessing) to assess its relative contribution to the complete model. This resulted in a five-parameter vMMM (presented in Töpfer, Barbieri, Soch, Bogler, & Haynes, 2020; for details, see the Appendix section: “von Mises mixture model”):

(2) $$\begin{array}{ccc} \;p\left(\theta_{i}|r_{1},r_{2},\mu ,\;\kappa_{1},\;\kappa_{2}\right) & = & r_{1}\cdot \mathrm{vM}\left(\theta_{i};0^{\circ }+\mu ,\kappa_{1}\right)\\ & & +\;r_{2}\cdot \mathrm{vM}\left(\theta_{i};180^{\circ }+\mu ,\kappa_{2}\right)\\ & & +\;r_{3}\cdot U\left(\theta_{i};0^{\circ },\;360^{\circ }\right). \end{array}$$

This model was estimated separately for each level of coherence for each participant. Note that, in a continuous circular space, a vMMM is required rather than a more conventional Gaussian mixture model (Bae & Luck, 2018, 2019; Barbieri et al., 2018). The vMMM was estimated using maximum likelihood estimation using a refined grid search (for details, see the Appendix section, Model estimation).

### Parameter estimation

At the first stage, we wanted to assess whether the model is plausible for the full range of experimental parameters that were tested. This step is vital, because the contribution of single components of the full vMMM can change drastically depending on the experimental condition. For example, at 100% coherence (pure signal condition), it would be expected that subjects were primarily able to detect the stimulus and place guesses only occasionally, say, because of distraction. Instead, at 0% coherence (pure noise condition), subjects were only guessing because there was no signal to be detected. But if the stimulus direction is in principle undetectable, contributions of detections and ROODs would be unexpected. Given that for 0% coherency there is no stimulus information, it is impossible for observers to guess the direction. In contrast, guesses should be only rare for the 100% stimuli. Thus, one could ask why we included these conditions in our model comparison. Our strategy was, however, not to fix the model decision a priori, but to let this result emerge from the model comparison stage. This strategy constitutes an additional sanity check of the modeling approach, in that it correctly eliminates model components from a condition where we have strong reasons to believe they will not exist.

The applicability of the model was assessed by using a formal model comparison approach, comparing Akaike weights computed from Akaike's information criterion (Akaike, 1974; Burham & Anderson, 2002; Wagenmakers & Farrell, 2004) of the full vMMM (containing all three components) and three different reduced versions of the vMMM (consisting of only one or two components). The reduced versions were created by setting the mixture coefficient (*r_i_*) of a specific component to zero, resulting in four different models: *m*_00_ (*r*_1_ =  *r*_2_ = 0; i.e., only guessing), *m*_01_ (*r*_1_ =  0; i.e., only guessing and ROOD), *m*_10_ (*r*_2_ = 0; i.e., only guessing and detection), and *m*_11_ (the full model). These models were chosen to test specific assumptions: *m*_00_ (only guessing) should perform well at 0% coherence; *m*_01_ (guessing and ROOD) would suggest that ROODs could appear even without detections (considered highly implausible a priori); *m*_10_ (guessing and detection) should perform well at high coherence conditions if no ROODs were present; *and* *m*_11_ (the full model) should perform well on high coherence conditions where ROOD responses were present (for details, see the Appendix sections, Model space and Model comparison).

As described elsewhere in this article, one or the other model might be more applicable to fit the distribution of response deviations depending on the coherence level. Therefore, the underlying model parameters were not estimated using a single model for all coherence levels. Instead, we estimated all models and used a model averaging approach to obtain average estimates of each parameter by calculating the weighted sum of the model-wise parameter estimates (${\hat{\phi }}_{i}$) across all four models (Burnham & Anderson, 2002) using the following function:

(3) $${\hat{\phi }}_{\mathrm{MA}}=\sum_{i=1}^{M}{\hat{\phi }}_{i}\cdot p\left(m_{i}|y\right).$$

In this equation, *p*(*m_i_*|*y*) denotes the posterior model probabilities for which Akaike weights were used in model averaging (Wagenmakers & Farrell, 2004). Model-averaged parameter estimates are likely to be more precise than estimates obtained using the same model for all coherence levels, because modeling uncertainty is considered in the estimation process (for details, see the Appendix section, Model averaging). For example, in very low coherence cases (e.g., 0%), parameter estimates should be dominated by the reduced model *m*_00_. Conversely, in very high coherence situations (e.g., 100%), parameter estimates should be dominated by *m*_10_ or *m*_11_, depending on whether there is evidence for ROODs (*m*_11_) or not (*m*_10_).

The analysis scripts as well as the related data are available at https://github.com/JoramSoch/RDK_vMMM.

### Statistical analyses

Statistical analyses were performed on the dependent variables (e.g., RT) and the model-averaged parameter estimates from the vMMM by computing separate mixed-effects repeated-measures analyses of variance (ANOVAs), with coherence as within-subject factor and RDK stimulus as well as response method as between-subject factors. To assess the source of significant main effects, we performed post hoc pairwise comparisons. The obtained results were Bonferroni corrected for multiple comparisons. To further evaluate the difference between response methods, we performed a mixed-effect ANOVA for each response method separately, with coherence as a within-subject factor and stimulus as a between-subject factor. To identify the sources of main effects, post hoc ANOVAs were performed for each stimulus type with coherence as a within-subject factor. For the evaluation of RT and response frequency, all coherence levels were used. For the statistical evaluation of the precision and bias parameter of detection and ROOD responses of the vMMM, only 12.5%, 25%, 50%, and 100% coherence were taken into account. This strategy was based on the fact that, at 0% coherence, no stimulus information was present, and subjects could only be guessing. Therefore, the estimated precision and bias parameter of detections and ROOD responses cannot be interpreted.

We applied a Greenhouse–Geisser correction when Mauchly's test indicated a violation of sphericity (*p* < 0.001). The significance threshold was a *p* value of less than 0.05. All the statistical analyses were performed using SPSS Version 25 (IBM, Armonk, NY).

## Results

### Presented versus reported motion directions

**Figure 3.**
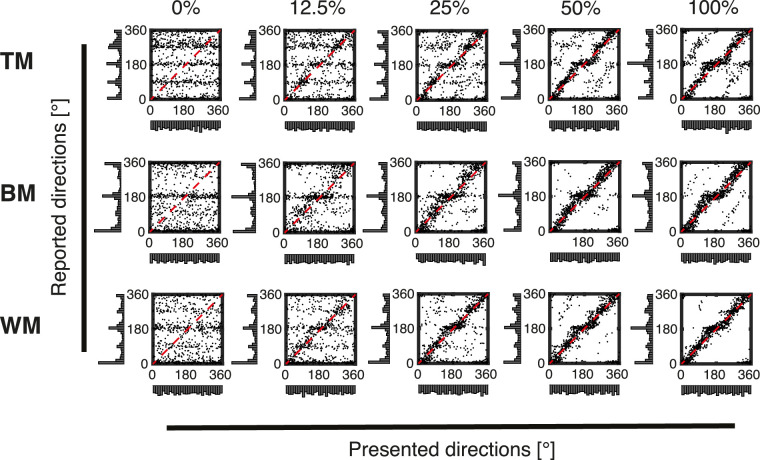
Results of trackball conditions. Scatterplots of trial-wise responses for all coherence levels (0%, 12.5%, 25%, 50%, and 100%) and stimulus types (TM, BM, and WM) collapsed across all subjects recorded with the trackball. In each panel, reported directions (*y*-axis) are plotted against presented directions (*x*-axis). Marginal distributions are shown as histograms. The dashed red diagonal displays the identity indicating optimal correspondence between stimulus and response. Note the horizontal stripes in the low coherency conditions indicating biases toward certain reported directions. In the high coherency conditions the biases can also clearly be seen as kinks. Also note the parallel lines that can be seen besides the main diagonal in the top two high coherency conditions. They indicate systematic misperception 180° opposite to the true direction (ROOD).

**Figure 4.**
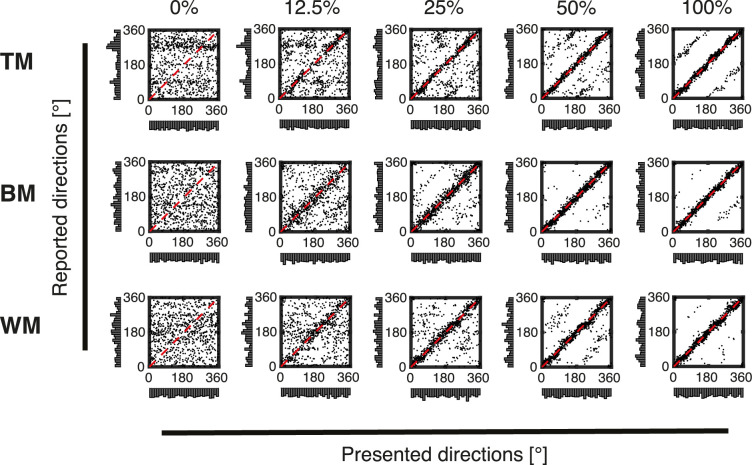
Results of rotating bar conditions. Scatterplots of trial-wise responses for all coherence levels (0%, 12.5%, 25%, 50%, and 100%) and stimulus types (TM, BM, and WM) recorded with the rotating bar. In each panel, reported directions (*y*-axis) are plotted against presented directions (*x*-axis). Marginal distributions are shown as histograms. The dashed red diagonal displays the identity indicating optimal correspondence between stimulus and response. Note the differences in biases for the low coherency conditions (especially the near absence of biases in the BM and WM conditions). Also note the parallel lines to the main diagonal in the top two high coherency conditions indicating reports that are offset exactly 180° from the true direction (ROOD). Compared with the trackball response condition (Figure 3) the biases are overall strongly decreased.

Figures 3 and 4 show the distribution of single trial responses separately for each experimental group, and coherence collapsed across all participants (Figure 3 for the trackball and Figure 4 for the rotating bar groups). The graphs plot the reported directions (*y*-axis) against the presented directions (*x*-axis) and the respective marginal distributions. The marginal distributions of the physical stimulus directions followed a uniform distribution in accordance with our experimental design that uniformly sampled the presented directions (Figures 3 and 4, marginal distributions on *x*-axis). Note that the slight deviations from uniformity are due to the fact that the uniform distribution is sampled with a limited number of trials. If we had an infinite number of trials, the *x*-axis marginals would converge toward uniformity.

At 100% coherence, there was a high correspondence between physical stimulus directions and reports across all stimulus and report conditions (Figures 3 and 4, scatterplots on the fifth column). This correspondence decreased with decreasing coherence (Figures 3 and 4, scatterplots from 0% to 50% coherence).

The marginal distributions of the responses (*y*-axis) obtained using the motor frame of reference (trackball; see the *y*-axis marginal distributions in Figure 3) deviated substantially from uniformity with high frequencies of responses at the cardinal directions 0° (up) and 180° (down) and especially for TM also at 90° (right) and 270° (left). In contrast, marginal distributions of responses obtained using a perceptual frame of reference (rotating bar, Figure 4 marginal distributions on the *y*-axis) approximate uniform distributions for most conditions. The exception being TM, where the distribution at low coherence levels seemed to be bimodal, with peaks centered around 90° (right) and 270° (left).

The correspondence between the presented stimulus direction and recorded responses was assessed using a Cramér von Mises test (for details see the Materials and Methods section, Target and response distributions). For the trackball response (Figure 3), the results suggested that the distribution of reported directions was significantly different from the distribution of presented directions for all stimulus types and at all coherence levels, including 100% coherence (Table 1).

**Table 1.** Cramér von Mises test statistics (*T*) for responses recorded with the trackball. *Notes:* The table presents the results of the test statistic to assess the difference between the distribution of presented motion and directions and the distribution of recorded motion directions for all coherence levels (0%, 12.5%, 25%, 50%, 100%) and stimulus types (TM, BM, and WM). *p < 0.05, **p < 0.01, ***p < 0.001.

	0%	12.5%	25%	50%	100%
TM	*T* = 1.17 *p* < 0.001***	*T* = 0.95 *p* = 0.006**	*T* = 1.01 *p* = 0.006**	*T* = 0.73 *p* = 0.011*	*T* = 1.10 *p* = 0.002**
BM	*T* = 4.87 *p* < 0.001***	*T* = 2.58 *p* < 0.001***	*T* = 2.85 *p* < 0.001***	*T* = 0.90 *p* = 0.007**	*T* = 0.82 *p* = 0.008**
WM	*T* = 4.38 *p* < 0.001***	*T* = 3.58 *p* < 0.001***	*T* = 1.94 *p* < 0.001***	*T* = 0.54 *p* = 0.031*	*T* = 0.94 *p* = 0.006**

For responses recorded with the rotating bar (Figure 4), the results of the Cramér von Mises test suggested that the distribution of reported directions was not significantly different from the distribution of presented directions at 100% coherence (Table 2). For TM, the divergence became significant starting from 50% coherence and increased between 25% and 0% coherence with decreasing coherence. For BM, there is no substantial difference between presented and recorded motion directions except at 12.5% coherence. For WM, the estimated divergence was significantly different at the lowest coherences (0% and 12.5%).

**Table 2.** Cramér von Mises test statistics (*T*) for responses recorded with the rotating bar. *Note*: The table presents the test statistic to assess the difference between the distribution of presented motion directions and the distribution of recorded motion directions for all coherence levels (0%, 12.5%, 25%, 50%, and 100%) and stimulus types (TM, BM, and WM). *p < 0.05, **p < 0.01, ***p < 0.001.

	0%	12.5%	25%	50%	100%
TM	*T* = 1.28 *p* < 0.001***	*T* = 0.63 *p* = 0.019*	*T* = 0.49 *p* = 0.042*	*T* = 0.62 *p* = 0.021*	*T* = 0.15 *p* = 0.401
BM	*T* = 0.30 *p* = 0.136	*T* = 0.52 *p* = 0.036*	*T* = 0.13 *p* = 0.444	*T* = 0.10 *p* = 0.578	*T* = 0.11 *p* = 0.527
WM	*T* = 0.77 *p* = 0.009**	*T* = 0.59 *p* = 0.024*	*T* = 0.23 *p* = 0.217	*T* = 0.25 *p* = 0.186	*T* = 0.10 *p* = 0.562

### RTs

**Figure 5.**
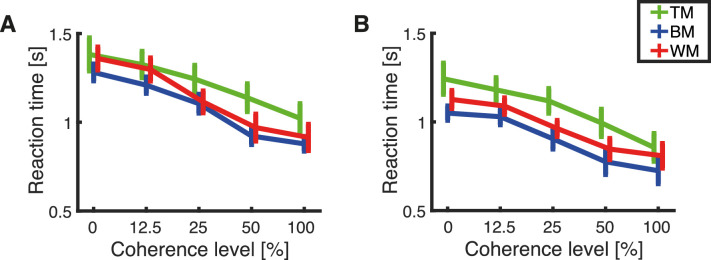
Mean RT of each group of subjects presented with TM (green), BM (blue), or WM (red). Error bars represent standard error of the mean. (A) Mean RT for responses recorded with the trackball. (B) Mean RT for responses recorded with the rotating bar.

RTs are plotted in Figure 5. As expected, RTs decreased with increases in coherence level for all combinations of stimulus and response methods (Figures 5A and 5B). A mixed effect repeated measured ANOVA unveiled a main effect of coherence level on RT, *F*(1.39, 91.57) = 146.35, *p* < 0.001 (Greenhouse–Geisser correction for sphericity). RTs differed between stimuli, as indicated by a main effect of stimulus on RT, *F*(2, 66) = 3.88, *p* = 0.028 (Greenhouse–Geisser correction for sphericity). Pairwise comparisons between stimuli revealed that this effect is mainly driven by the difference in RTs between TM and BM (*p* = 0.023, Bonferroni-corrected for multiple comparisons). In contrast, the pairwise comparison between TM and WM (*p* = 0.269, Bonferroni-corrected for multiple comparisons) and WM and BM (*p* = 0.912, Bonferroni-corrected for multiple comparisons) did not reveal any significant differences. The main effect of response methods on the RT, *F*(1,66) = 11.55, *p* = 0.001, depicts a significant difference in RTs between responses recorded with the trackball and those recorded with the rotating bar. A pairwise comparison unveiled that responses obtained with the trackball were slower than responses recorded with the rotating bar (*p* = 0.001, Bonferroni-corrected for multiple comparisons).

### Distribution of response deviations

**Figure 6.**
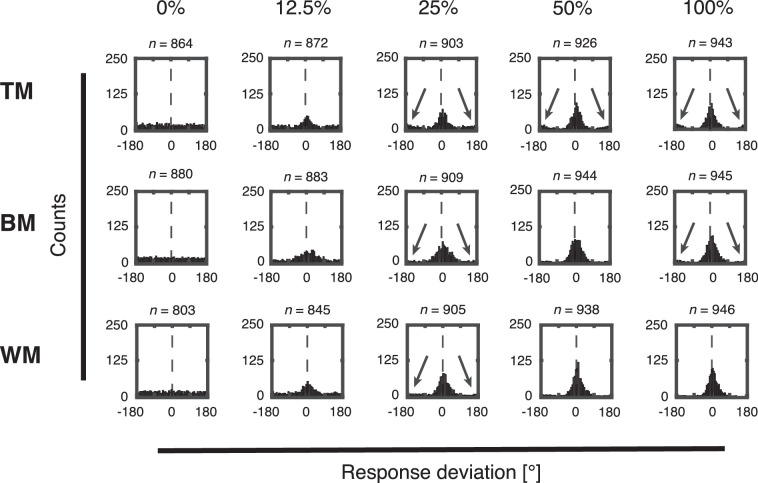
Histogram of trial-wise response deviations separately for each coherence level (0%, 12.5%, 25%, 50%, and 100%) and stimulus type (TM, BM, and WM) collapsed across all subjects recorded with the trackball. Vertical dashed lines center on Δθ = 0° to indicate the correct choice. Black arrows indicate clusters of responses that were placed in the opposite direction (ROOD).

**Figure 7.**
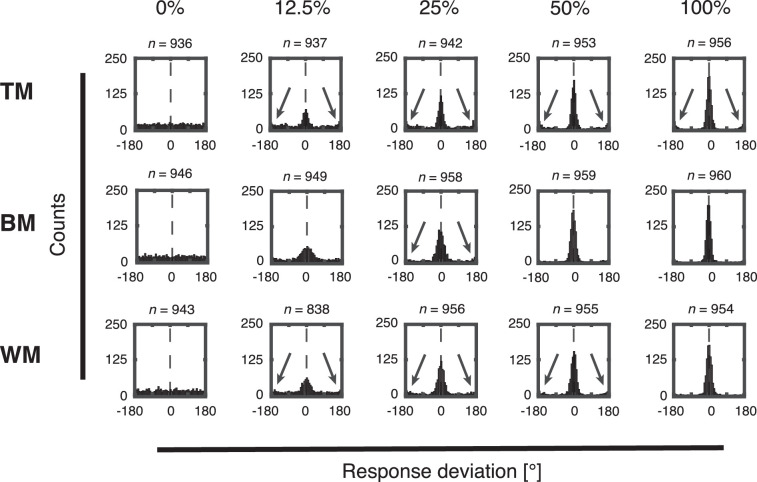
Histogram of trial-wise response deviations separately for each coherence level (0%, 12.5%, 25%, 50%, and 100%) and stimulus types (TM, BM, and WM) collapsed across all subjects recorded with the rotating bar. Vertical dashed lines center on Δθ = 0° to indicate the correct choice. Black arrows indicate clusters of responses that were placed in the opposite direction (ROOD).

We also computed single trial response deviations as the difference between presented and reported motion direction. These trial-wise deviations were collapsed across directions and subjects for each experimental group (Figures 6 and 7). At 100% coherence, when the stimulus does not contain noise, subjects responded precisely and response deviations cluster around Δθ = 0° for all stimuli (Figures 6 and 7, 100% coherence; but see Figure 13 for a model-based analysis revealing subtle biases). As coherence levels decrease, the response deviations still cluster around Δθ = 0°, but the precision decreases as well for all stimuli (Figures 6 and 7, 12.5%–50.0% coherence).

Interestingly, subjects responded in the opposite direction (±180° relative to the true direction) in a consistent proportion of trials, as reported previously (Bae & Luck, 2018; Barbieri et al., 2018). We call this a ROOD (indicated with black arrows in Figures 6 and 7). At 0% coherence, no net motion direction was presented on the screen. Thus the distributions of response deviation were uniformly spread and no ROOD responses were visible for all stimuli, as expected (Figures 6 and 7, 0% coherence). These ROODs are also apparent in the original distributions of presented versus reported stimulus directions as the off-diagonal responses in Figures 3 and 4 (best visible in the 100% coherence condition).

### Model comparison

**Figure 8.**
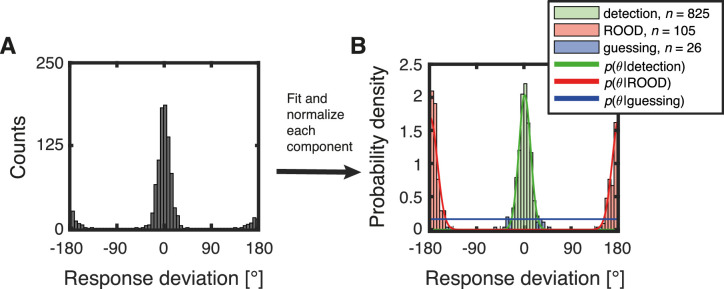
Exemplary application of the five-parameter vMMM. (A) Distribution of response deviations obtained by concatenating single trial response deviations of all subjects stimulated with TM at 100% coherence using the rotating bar. The model was fit to the data by optimizing parameters using maximum likelihood estimation. (B) For better visualization, the data of the components 1) detection in green, 2) report in the opposite direction (ROOD) in red, and 3) guessing in blue were normalized and the corresponding probability density functions plotted as well. Note that this figure does not represent the model estimation process, but displays the individual normalized components and their contribution to the vMMM.

Based on the distribution of response deviations (Figures 6 and 7), we chose a model with a mixture of three components: 1) detection, 2) ROOD, and 3) guessing (for details see the Materials and Methods section, Modeling behavioral performance). To evaluate this hypothesis, we fitted different versions of the five-component vMMM to the distributions of response deviations. We assessed the relative quality of each model fit by using an estimator of prediction error (Akaike information criterion weights) in the course of a model assessment procedure (for details, see the Appendix sections, Model space, Model estimation, and Model comparison). The higher the score of the Akaike information criterion weights, the more information the model conveys about the underlying distribution. Figure 8 presents an example of the vMMM model fitted on the distribution of response deviations obtained using TM at 100% coherence recorded with the rotating bar.

**Figure 9.**
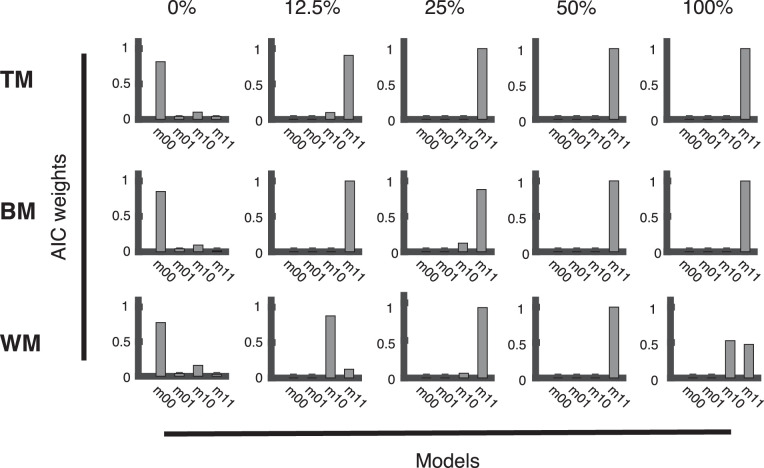
Model comparison for trackball conditions. Bar plots presenting the Akaike information criterion (AIC) weights for all stimulus types (TM, BM, and WM) and coherence levels (0%, 12.5%, 25%, 50%, and 100%) to evaluate the fit of four different vMMMs on the distribution of response deviations pooled across all subjects responding with the trackball. The full model (*m*_11_) consists of a vM to fit detections, a vM to fit reports of the opposite direction (ROOD), and a uniform distribution to fit guesses. Three reduced versions of the full model are presented as well: *m*_01_ allows to fit ROODs and guesses; *m*_10_ allows to fit detections and guesses; *m*_00_ only allows fitting guesses.

**Figure 10.**
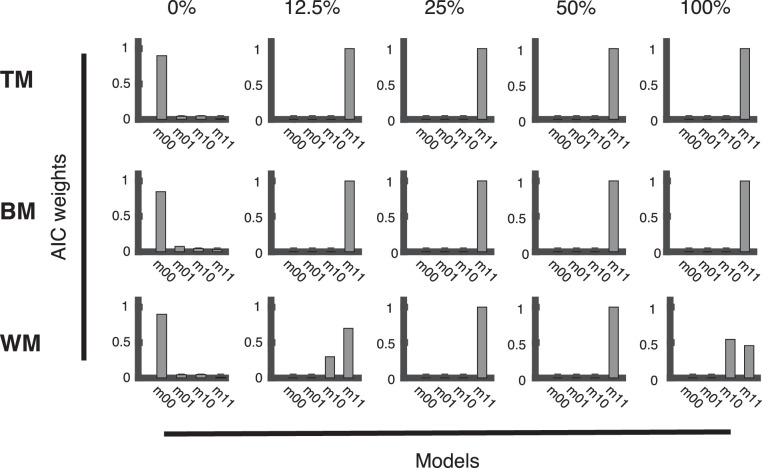
Model comparison for rotating bar conditions. Bar plots presenting the Akaike information criterion (AIC) weights for all stimulus types (TM, BM, and WM) and coherence levels (0%, 12.5%, 25%, 50%, and 100%) to evaluate the fit of four different vMMMs on the distribution of response deviations pooled across all subjects responding with the trackball. The full model (*m*_11_) consists of a vM to fit detections, a vM to fit reports of the opposite direction (ROOD), and a uniform distribution to fit guesses. Three reduced versions of the full model are presented as well: *m*_01_ allows to fit ROODs and guesses; *m*_10_ allows to fit detections and guesses; *m*_00_ only allows fitting guesses.

Akaike weights obtained during model comparison can be seen as the relative likelihoods of each model, that is, the conditional probability that this model describes the generating process, given the data from a given coherence level and the set of candidate models (for details, see the Appendix section, Model comparison). The plots of the Akaike's information criterion weights (Figures 9 and 10) showed a very similar pattern for responses recorded with the trackball (Figure 9) and responses recorded with the rotating bar (Figure 10). The full model (*m*_11_) elicited the best fit for the distributions of response deviations obtained with TM and BM stimulation for all coherences, except 0% and for both response methods (Figures 9 and 10). For WM, this was true as well, except for 12.5% and 100% coherence (Figures 9 and 10, WM). In this condition, a reduced model (*m*_10_) that fits detections and guesses, but no ROOD responses described the data sufficiently well (Figures 9 and 10, WM). Importantly, as expected, at 0% coherence, when no stimulus information was present, the optimal model fit was obtained by the model that only contains a uniform distribution guessing component for all stimuli (*m*_00_, Figures 9 and 10).

### Detection frequency

**Figure 11.**
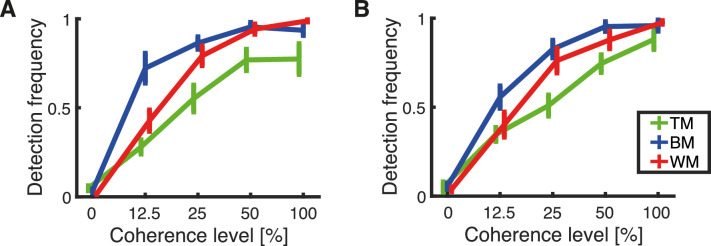
Estimated means of detection frequency calculated separately for each subject. The plots show the dependency of the detection frequency on the level of coherence for each stimulus type, TM (green), BM (blue), and WM (red). Error bars represent the standard error of the mean. The results are presented for (A). Responses obtained with the trackball and (B) responses obtained with the rotating bar.

The detection frequency denotes the fraction of all trials that are estimated as detections by the model (Figure 11). Note that the assumption underlying the model is that the participant either detects the stimulus with a certain precision, reports an opposite direction, or guesses. Thus, performance can be improved by increasing the number of detections or by increasing the precision for the detections. The detection frequency is, therefore, independent of the precision of the detection distribution (reported separately elsewhere in this article). There was an increase in detections with coherence for both response methods. The number of detections plateaus at approximately 50% coherence for responses reported with the trackball using BM and TM, whereas it seemed to increase further for WM at 100% coherence (Figure 11A). For responses recorded with the rotating bar, the number of detections plateau in the BM condition at 50%, while they increased for TM and WM at 100% coherence (Figure 11B).

A mixed-effects repeated-measures ANOVA showed a main effect of coherence on detection frequency, *F*(2.99, 197.26) = 633.89, *p* < 0.001 (Greenhouse–Geisser correction for sphericity). A post hoc pairwise comparison revealed a significant increase in detection frequency for each coherence from 0% to 50% (pairwise comparison *p* < 0.001, Bonferroni corrected for multiple comparisons), but not between 50% and 100% (*p* = 0.074, Bonferroni corrected for multiple comparisons). The RDK type had a main effect on detection frequency, *F*(2, 66) = 14.90, *p* < 0.001, as well. A post hoc pairwise comparison between stimulus types revealed that TM elicits fewer detections than BM (*p* < 0.001, Bonferroni corrected for multiple comparisons) and WM (*p* = 0.002, Bonferroni corrected for multiple comparisons), although no difference between BM and WM was found (*p* = 0.235, Bonferroni corrected for multiple comparisons). There was no main effect of response methods on detection frequency, *F*(1, 66) = 0.15, *p* = 0.704.

### Detection precision

**Figure 12.**
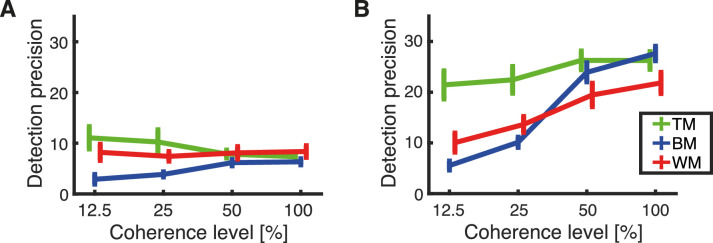
Estimated means of detection precision (κ_1_) collapsed across subjects. The plots show the dependency of the detection precision on the level of coherence for each stimulus type: TM (green), BM (blue), and WM (red). Error bars represent the standard error of the mean. (A) Responses obtained with the trackball. (B) Responses obtained with the rotating bar.

Detection precision refers to the width of the distribution of response deviations (Figure 12; this is akin to width of the green distribution in Figure 8, right) and is represented in the model by the parameter kappa which is the reciprocal of the variance. The higher the kappa value, the narrower the distribution and the better the performance. For a uniform distribution, akin to guessing, the kappa would reach its smallest value, 0.

The detection precision (κ_1_) for responses recorded with the trackball was in the range of kappa between 2 and 10 and maintained stable throughout different levels coherence for all RDK types (Figure 12A). Note that a constant precision across coherence levels is still compatible with increased performance with higher coherence because the detection frequency (differently from guessing) increases with coherence (Figure 11). The estimated detection precision for responses recorded with the rotating bar for the same stimuli was consistently higher (kappa range between 5 and 30; i.e., more precise) compared with responses recorded with the trackball. Although the estimated detection precision obtained using the rotating bar and TM seemed to stay constant throughout coherence levels, precision for BM and WM increased with increasing coherence level (Figure 12B). Here, the higher sensibility of the rotating bar reports possibly allowed to reveal these additional gradual increases.

A mixed-effects repeated-measures ANOVA performed for all coherence levels except 0% (which is a special case because it is expected to follow a uniform distribution) showed a main effect of coherence, *F*(3, 198) = 37.26, *p* < 0.001, and RDK type, *F*(2, 66) = 12.51, *p* < 0.001, on detection precision. Both effects were primarily driven by responses recorded from the groups of participants responding with the rotating bar, as suggested by Figure 12 and indicated by a main effect of response method, *F*(2, 66) = 139.22, *p* < 0.001, on detection precision. This is underlined by a post hoc pairwise comparison between response methods that revealed a significantly higher precision obtained with the rotating bar compared with the trackball responses (*p* < 0.001).

To assess the difference between response methods, we performed an additional mixed-effects ANOVA for each response method separately, with coherence as a within-subject factor and stimulus as a between-subject factor. For responses recorded with the trackball (Figure 12A), there was no main effect of coherence on detection precision, *F*(1.96, 64.53) = 0.03, *p* = 0.974 (Greenhouse–Geisser correction for sphericity), but a main effect of stimulus type, *F*(2, 33) = 7.54, *p* = 0.002. A post hoc pairwise comparison between all stimuli allowed to determine a significantly higher detection precision for TM than BM (*p* = 0.002, Bonferroni corrected for multiple comparisons) and a higher detection precision for WM than BM (*p* = 0.027, Bonferroni-corrected for multiple comparisons). An additional repeated-measures ANOVA with coherence as within-subject factor, performed separately on each RDK type, did not reveal a main effect of coherence on detection precision for TM, *F*(3, 19.34) = 1.15, *p* = 0.33 (Greenhouse–Geisser correction for sphericity) and WM, *F*(3, 16.59) = 0.22, *p* = 0.744 (Greenhouse–Geisser correction for sphericity), but a main effect of coherence on detection precision for BM, *F*(3, 33) = 14.83, *p* < 0.001.

For responses obtained with the rotating bar (Figure 12B), there was a main effect of coherence on detection precision, *F*(3, 99) = 63.07, *p* < 0.001. Furthermore, there was a main effect of RDK type on detection precision, *F*(2, 33) = 8.37, *p* < 0.001. A post hoc pairwise comparison between all stimuli revealed a significantly higher detection precision for TM than BM (*p* = 0.005, Bonferroni corrected for multiple comparisons) and for TM than WM (*p* = 0.003, Bonferroni corrected for multiple comparisons), although BM and WM did not show difference in mean detection precision (*p* = 1, Bonferroni corrected for multiple comparisons). An additional repeated-measures ANOVA with coherence as within-subject factor, performed on each RDK type separately, revealed a main effect of coherence on detection precision for BM, *F*(3, 33) = 90.36, *p* < 0.001, and WM, *F*(3, 33) = 17.82, *p* < 0.001. Interestingly, we did not find a main effect of coherence on detection precision for TM, *F*(3, 33) = 2.51, *p* = 0.098 (Greenhouse–Geisser correction for sphericity).

### Biases: Detection distribution mean

Systematic shifts in the mean (or the center) of the distributions of response deviations can be informative about potential systematic biases, for example, in the form of a rotation of the motor coordinate frame. This would happen if participants consistently rotate the trackball reports relative to the main axes, for example. Note that such a rotation does not, in itself, predict a deviation from uniformity of the reports. Instead, it is independent from the biases toward the cardinal directions observed in Figures 3 and 4. Here, a clockwise shift from Δθ = 0° of the mean response deviations distribution can suggest that subjects consistently respond clockwise to the presented direction of the stimulus throughout all trials. Note that we only assessed this parameter for the distribution of response deviations of detections. It would be possible to estimate the mean (or the center) of the response deviations distributions of ROOD responses as well, incorporating another free parameter in the vMMM, but this would have been based on far fewer cases. Thus, we did not perform such an analysis.

**Figure 13.**
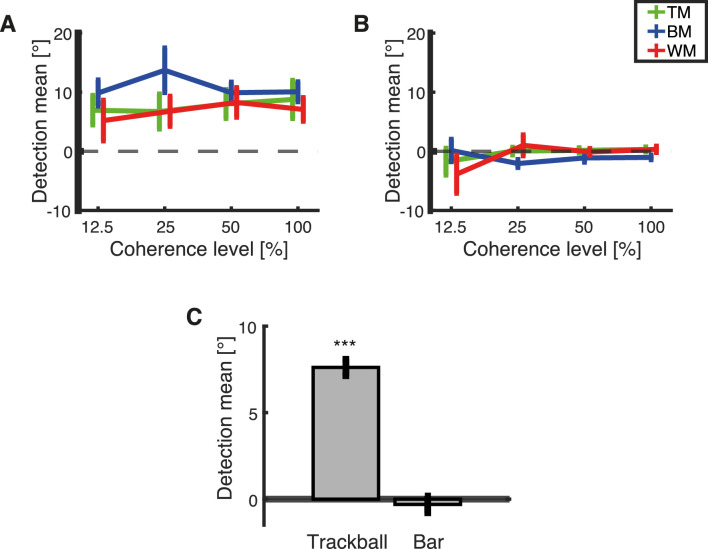
Estimated means of detection distributions calculated separately for each subject. The plots show the dependency of the mean of the detection distributions on the level of coherence for each stimulus type: TM (green), BM (blue), and WM (red). Error bars represent the standard error of the mean. The results are presented for (A) responses obtained with the trackball and (B) responses obtained with the rotating bar. (C) Overall average of the mean center of the distribution of detections for trackball and rotating bar. Estimated by averaging the bias of all subjects, all coherence levels and all RDKs for each response method. The asterisk indicates a significant shift from the center of correct responses (Δθ = 0°), tested by a one-sample two-tailed *t*-test. ***p < 0.001.

The group-level mean of the estimated detections is presented in Figure 13. For responses recorded with the trackball, the mean of the response deviations distribution was consistently shifted around 10° clockwise from Δθ = 0°. This factor was consistent throughout different levels of coherence and types of stimuli (Figure 13A). Detection responses recorded with the rotating bar primarily center around Δθ = 0° for all coherence level and stimuli (Figure 13B).

A mixed-effect repeated-measures ANOVA including all coherence levels except 0% did not reveal any main effect of coherence, *F*(1.84, 121,47) = 1.40, *p* = 0.245 (Greenhouse–Geisser correction for sphericity) or RDK type, *F*(2, 66) = 0.66, *p* = 0.519, but a main effect of response method, *F*(1, 66) = 46.03, *p* < 0.001 (Figure 13). We wanted to understand whether there were substantial offsets from 0° inherent in each of both response methods. Therefore, we collapsed the estimated mean of all coherences and stimuli separately for each response method and calculated the overall average (Figure 13C). Separate two-tailed *t*-tests for each response method determined a substantial offset of the mean center of detection distributions, *t*(143) = 9.05, *p* < 0.001, for responses recorded with the trackball (*M* = 6.58°, *SE* = 0.73°), whereas for the rotating bar (*M* = –0.66°, *SE* = 0.41°), there is no significant offset from Δθ = 0°, *t*(143) = –1.60, *p* = 0.113. The clockwise shift for trackball responses was not visible by eye inspecting distributions of response deviations (Figure 6)

### Systematic misperception: ROOD frequency

**Figure 14.**
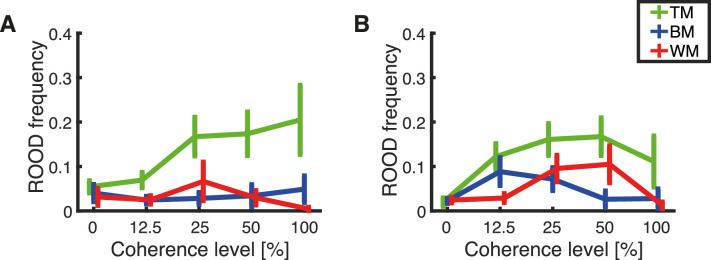
Estimated means of the frequency of ROOD calculated separately for each subject. The plots show the dependency of the ROOD frequency on the level of coherence for each stimulus type, TM (green), BM (blue), and WM (red). Error bars represent the standard error of the mean. The results are presented for (A) responses obtained with the trackball and (B) responses obtained with the rotating bar.

The ROOD frequency is the estimated fraction of responses exactly 180° opposite to the true direction (Figure 14). Thus, in contrast to the guesses, these are systematic misperceptions. We found considerable variation in the estimated proportion of ROOD between modes of report and stimulus types. Interestingly, TM seemed to induce the highest number of ROOD responses independent of response method and coherence. WM, in contrast, had very few ROOD responses at 100% coherence, although it exceeded the number of ROOD responses estimated for BM at a medium coherence level (25%). Note that as expected at 0% coherence there were very few ROOD responses, because there was no information presented and the subjects were only guessing.

A mixed-effect repeated-measures ANOVA revealed a main effect of coherence on ROOD frequency, *F*(2.97, 195.75) = 5.29, *p* = 0.002 (Greenhouse–Geisser correction for sphericity). Additionally, we found a main effect of RDK type on ROOD frequency, *F*(2, 66) = 14.46, *p* < 0.001. A post hoc pairwise comparison between stimulus types revealed a higher ROOD frequency for TM compared with BM (*p* < 0.001, Bonferroni corrected for multiple comparisons) and TM compared with WM (*p* < 0.001). No difference was found between BM and WM (*p* = 1, Bonferroni corrected for multiple comparisons). There was no main effect of the response method on ROOD frequency, *F*(1, 66) = 0.144, *p* = 0.706.

### ROOD precision

**Figure 15.**
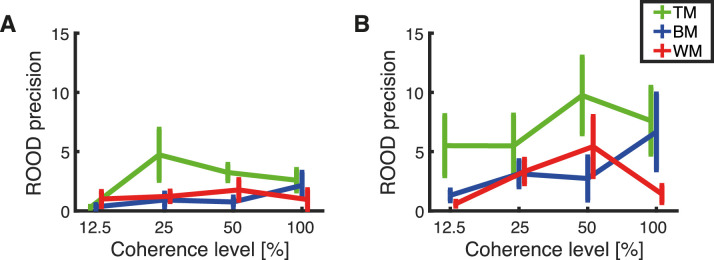
Estimated means of the precision of ROOD calculated separately for each subject. The plots show the dependency of the ROOD precision (κ_2_) on the level of coherence for each stimulus type, TM (green), BM (blue), and WM (red). Error bars represent the standard error of the mean. The results are presented for (A) responses obtained with the trackball and (B) responses obtained with the rotating bar.

ROOD precision (κ_2_) refers to the density parameter of the response deviations distribution that clustered around 180° from the presented direction (Figure 15). ROOD responses recorded with TM had consistently higher ROOD precision compared with BM and WM independent of response method and coherence level. We found a lower precision estimate of ROOD responses when subjects reported with the trackball (Figure 15A) compared with the other group of participants that have been responding with the rotating bar (Figure 15B).

A mixed-effects ANOVA on all coherence levels except 0% determined a significant effect of coherence on ROOD precision, *F*(2.39, 157.68) = 3.16, *p* = 0.037 (Greenhouse–Geisser correction for sphericity) and a significant effect of RDK type, *F*(2, 66) = 5.06, *p* = 0.009. Further, a main effect of response method on ROOD precision was evident, *F*(1, 66) = 10.92, *p* = 0.002. A post hoc pairwise comparison between response methods revealed a higher ROOD precision for the subjects responding with the rotating bar than for those responding with the trackball (*p* = 0.002, Bonferroni corrected for multiple comparisons). Two separate mixed-effects ANOVAs were performed for each response method separately, with coherence as a within-subject factor and stimulus as a between-subject factor. In this case, we found no main effect of any of the factors used in the analysis.

### Guess frequency

**Figure 16.**
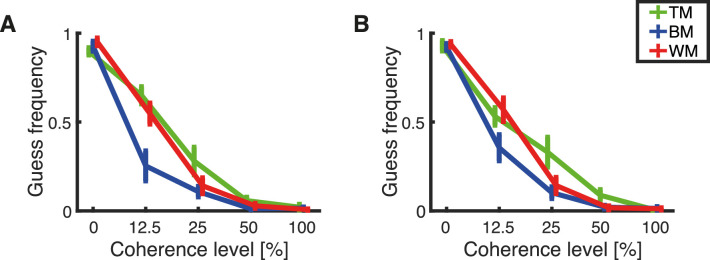
Estimated means of the frequency of guesses calculated separately for each subject. The plots show the dependency of the guess frequency on the level of coherence for each stimulus type, TM (green), BM (blue), and WM (red). Error bars represent the standard error of the mean. The results are presented for (A) responses obtained with the trackball and (B) responses obtain with the rotating bar.

The guess frequency was the fraction of all trials that were guesses according to the estimated model (Figure 16). The model estimates for guess frequency at 0% coherence are all larger than 0.9. The guess frequency consistently decreased with increasing coherence up to the point that there are almost no guesses at 100% coherence. BM seemed to induce the lowest amount of guesses independent of coherence level and response mode. There were no large differences between responses recorded with the trackball (Figure 16A) and responses recorded with the rotating bar (Figure 16B).

A mixed-effects repeated-measures ANOVA revealed a main effect of coherence on guess frequency, *F*(2.25, 148.61) = 672.26, *p* < 0.001 (Greenhouse–Geisser correction for sphericity). A post hoc pairwise comparison between coherence levels revealed that the guess frequency was different across all coherence levels (each pairwise comparison *p* < 0.003, Bonferroni corrected for multiple comparisons). There was a main effect of RDK type on guess frequency, *F*(2, 66) = 9.63, *p* < 0.001. A post hoc pairwise comparison between stimulus types revealed that TM leads to more guesses than BM (*p* < 0.001, Bonferroni corrected for multiple comparisons), but did not elicit more guesses than WM (*p* = 0.225, Bonferroni corrected for multiple comparisons). Furthermore, BM had fewer guess trials than WM (*p* = 0.039, Bonferroni corrected for multiple comparisons). There was no main effect of response method on guess frequency, *F*(1, 66) = 0.077, *p* = 0.782.

## Discussion

Our study assessed the contributions of perceptual and motor processes on judgments of feature-continuous motion stimuli. For this, we presented three different random dot motion stimuli and recorded responses using a motor frame of reference (trackball) and a perceptual frame of reference (rotating bar). Using computational modeling, we compared the parametric contributions of veridical detections, systematic misperceptions, and pure guesses. Our primary finding is that the perceptual frame of reference (rotating bar), which is free of directionally dependent motor processing, yields judgments that are more precise and less biased. We also compared different RDKs using a vMMM and found parametric differences between TM compared with BM and WM stimuli that were more similar.

### Effect of report method on judgments

The raw plots of report versus stimulus direction (Figures 3 and 4) reveal several anisotropies. To some degree, this finding reflects the effects of reporting, because the biases are much weaker for the visual comparison stimulus condition than for the trackball only condition (cf. Figure 3 vs. Figure 4). In the trackball condition (Figure 3) there is a strong tendency to report the up and down conditions and also under certain conditions (especially TM) to report the left and right conditions. This is not apparent to the same degree when using the visual comparison stimulus (Figure 4). Thus, this might partially reflect motor biases related to the ergonomics of the trackball (as discussed elsewhere in this article). Even the visual comparison stimulus condition has some anisotropies, especially for the TM condition (Figure 4, top row). This could reflect either the known anisotropies in motion perception, especially the decreased discriminability of motion directions at oblique angles (Gros, Blake, & Hiris, 1998). Also, this could reflect implicit assumptions or prior expectations that participants have about the probability of different stimuli. It would have been possible to address also these biases by developing a different model; however, note that our primary focus in this study was not the full distribution that exhibits these anisotropies, but the distribution of deviations from the veridical direction (Figures 6 and 7). Here, using the vMMM we found a clockwise shift of judgments recorded with the trackball (Figures 13A and C) that could also be circumvented using the rotating bar (Figures 13B and C). Additionally, it unveiled that responses recorded with the trackball were less precise (Figure 12A) than responses recorded with the rotating bar (Figure 12B).

Purely motor-based response methods require visuomotor transformations of the stimulus directions presented on the monitor onto directional motor movements of the limbs (for a review, see Colby, 1998). The graded movement of the limbs can be recorded using a response device such as a trackball, mouse, or joystick. The ergonomy of the limb movements, as well as the interplay with an analog device, can cause biases and motor variability that confounds behavior performance (Engen, 1971; Green & Swets, 1966; Menozzi et al., 2016; Smith, 2016). Presumably, the confounds in the trackball responses originate from the ergonomic properties of the hand movements and the relative position of the subject to the device. The trackball required the subject to give a response using the index and the middle finger. It is easier to move those fingers on their joints upwards or downwards than to, for example move them left or right. This factor might have caused the vertical bias (cf. Figure 3 and Table 1). If we had asked subjects to use an other digit for report (e.g., their thumb), this bias might have been different (Schütz, 2014). Our procedure explicitly aimed to prevent subject-specific rotational biases in the position of the hand on the trackball. For this, the relative position of subjects to the trackball was held constant by fixing the trackball on the desk using a strip of Velcro and placing the head of the subjects on a chin rest. It seems that this fixed relative position of the hand of all subjects on the trackball was nonetheless associated with a slight consistent rotation of the frame of reference for the trackball, which is expressed as a rotational bias in our model (cf. Figures 13A and C). Note that, in addition to these precautions, differences in individual ergonomic fit between trackball and the subjects’ hands might have caused further variations in the relative positioning. These factors might have additional contributed to the lower detection precision of the trackball compared with the rotating bar where the rotational bias is not evident (cf. Figure 12).

To place a response using the rotating bar, subjects had to compare the memorized direction of the moving dots to a probe stimulus: a rotating line presented in the same coordinate frame. Subsequently, they needed to press a single button when the probe stimulus overlapped with the direction of choice. Note that this also involves a transformation, but only within the visual modality—from the direction of the dots to the rotating bar—but no transformations between modalities were required. The actual movement was kept the same for each report; thus, motor-dependent directional error was excluded. The variance of the rotating bar judgments is thus independent of motor variation. Our data show that the advantages of using a perceptual comparison stimulus far outweigh any potential disadvantages (such as perceptual load, interference, or similar). The precision of responses given through perceptual comparison was considerably higher (compared with the motor response), and, interestingly, increased with motion coherence. Thus, the low precision and biases of the motor report might obscure such perceptual improvements.

One main difference between both response methods is the absence of visual feedback to guide the responses for the purely motor-based responses. Thus, the rotating visual comparison stimulus can decrease the variability because the subject can directly compare the match of the direction, but it involves an integration of sensory and motor control signals, thus potentially changing the duration of a response (Sülzenbrück & Heuer, 2011). Interestingly, although the increase in motor variability is obvious (Figure 12), we did not find an increased response time for responses using a visual cue (Figure 5). In the literature, there is another type of response, which we did not test in the current paper. In this adjustment method, subjects use a graded device (e.g., a trackball) to move a visible cursor in the direction of choice. Such responses are characterized by a mixed frame of reference, which combine a motor-based with a perceptual-based response (Bae & Luck, 2019; Bays, Catalao, R& Husain, 2009; Nichols & Newsome, 2002; Pilly & Seitz, 2009; Prinzmetal et al., 1997; Wilken & Ma, 2004; Zhang & Luck, 2008; Zokaei et al., 2011). It would be interesting to see whether these mixed reports are influenced by motor biases as well. To date, little attention has been paid to the impact of various report methods on behavior. More research is needed to fully unveil the relative contributions of sensory or motor contributions and their potential interactions in such experiments.

### Systematic misperception

Our feature-continuous task design revealed an additional type of judgment: a systematic misperception which we term “report in the opposite direction” (ROOD), and that has also been observed previously by us and others (Bae & Luck, 2018; Barbieri et al., 2018). Note that this perceptual phenomenon would have gone unnoticed in classical decision-making experiments with few discrete directional bins (e.g., motion left/right). These ROODs would have instead been confused with pure guesses. The systematic misperception is different from mis-binding errors presented in experiments about working memory (Zokaei et al., 2011). ROOD responses appeared consistently for all tested stimuli and report methods except at 0% coherence, when no motion information is presented and thus participants would have in principle not been able to guess the correct direction. This is suggested by our model comparison approach, where we compared fits of a model comprising detections, guesses and ROODs (*m*_11_, full model) against reduced versions of the model (cf. Figures 9 and 10). This analysis unveiled that 1) the model only estimates the presence of ROOD responses when stimulus information is actually presented, thus constituting a sanity check of the modeling (cf. Figures 9 and 10, reduced model *m*_00_: at 0% coherence a pure guessing model fits the data best); 2) no subject exclusively reported the opposite direction (cf. Figures 9 and 10, reduced model *m*_01_: a model consisting of ROOD and guesses only does not explain the data).

To understand the nature of the ROOD responses, we asked every subject after the experiment was completed whether they ever had the feeling of perceiving the axis of motion instead of the direction. Approximately two-thirds of the subjects (54/72) responded that they sometimes had the feeling of perceiving the axis of motion instead of perceiving a specific direction. Importantly, for our task we required participants to always place a response, even when no direction was perceived. We hypothesize that subjects might have used a guessing strategy when they perceived only the axis of motion instead of a dominant motion direction. Thus, we suggest that ROODs might be based on an independent information source, namely, the axis of motion. A response based on the axis of motion can yield a wrong report with a 50% chance. This incorrect report, in the opposite direction, is what we refer to as ROOD. Importantly, a guessing response based on a perceived axis of motion has a 50% chance of falling in the correct direction as well. Therefore, some of the detections might also be based on the motion axis and not on the detection of motion direction. Interestingly, ROOD precision seemed to be lower than detection precision for medium and high coherence levels (cf. Figures 12B and 15B).

A potential explanation for the perception of opposite direction could be the discrete nature of our stimulus steps in terms of frames and pixels. This could yield similar undersampling effects as in the wagon-wheel effect (Finlay, Dodwell, & Caelli, 1984). However, in the case of the wagon wheel, there are only two motion directions involved (veridical and opposite), whereas our motion stimuli covers the entire space of directions. Thus, for our stimuli it is not clear why this would selectively result in a motion experience that is exactly opposite to the veridical direction.

Despite the clear evidence for ROOD responses both from the data (Figures 3, 4, 6, and 7) and subjective reports, there were not sufficient trials to assess additional properties of these responses, especially whether they vary with direction. Further research would be needed to shine light on the nature of this phenomenon (Manning, Meier, & Giaschi, 2022), for example, by maximizing the number of ROOD by focusing on fewer conditions (Bae & Luck, 2022).

### Effects of motion type on continuous performance

We also compared the performance for three different versions of the RDK motion stimulus. Visual inspection of the accumulated trial-wise responses (Figure 4) suggested only minor differences between the motion displays. For TM at 0% and 12.5% coherence, a horizontal bias was evident (cf. 0% and 12.5% coherence levels in Figure 4A) that was not present for BM and WM (cf. 0% and 12.5% coherence levels in Figures 4B and C). The distributions of response deviations cluster around *∆θ* = 0° for all three stimuli (cf. Figures 7A, B, and C). According to the estimated model, TM responses were slower (cf. Figure 5B) and elicited fewer detections (cf. Figure 11B), but detections were more precise (cf. Figure 12B) compared with BM and WM. There was no significant difference between BM and WM in RT, number of detections, and detection precision. This finding might suggest that subjects choose a different trade-off between RT, detection, and precision for TM compared with BM and WM.

Interestingly, detection precision depended on coherence level for BM and WM, but it was independent of coherence level for TM (cf. Figure 12B). This outcome could be a consequence of different perceptual strategies to infer motion direction from TM compared with BM and WM stimuli, as suggested by research on smooth pursuit performance (Schütz et al., 2010). Schütz et al. (2010) suggested that the perceptual system integrates signal and noise information in BM and WM stimuli. Thus, the detection precision for these stimuli decreases with increasing noise. Instead, for TM, the perceptual system separates the stimulus in a signal and a noise surface. In this case, detection precision would be independent of the noise level. Importantly, the perceptual mechanism for detecting BM and WM stimuli is not fully equivalent. Importantly, the perceptual mechanism for detecting BM and WM stimuli is not fully equivalent according to Schütz et al. (2010). In accordance, Pilly and Seitz (2009) found considerable differences between BM and WM stimuli when changing stimulus parameters such as the spatiotemporal displacement and contrast of the dots, as well as the aperture size. However, using stimulus parameters optimized for detections—such as medium speed, high contrast, and long stimulus durations—Pilly and Seitz (2009) already found that the performance in response to BM stimuli does not differ strongly to the performance to WM stimuli, which is in line with our research.

Note that, at 100% coherency, the different stimulus types (TM, BM, and WM) are identical. Thus, this finding raises the question why there are performance differences between these identical conditions, with especially the performance for the TM condition being worse than for BM and WM (Figure 11A). Note that these performance differences for identical stimuli are only significant for the trackball condition, possibly reflecting its overall lower accuracy. A possible explanation of the different performance of these identical stimuli is that every participant saw only one type of stimulus and thus the other stimuli that every person judged were different. For example, an observer assigned to TM saw the same stimulus for 100% as a BM observer, but different stimuli for other coherency levels. Owing to these differences in temporal context, the participants might have adopted slightly different strategies for identifying their motion type, thus potentially causing the different performance for identical stimuli at 100%.

## Conclusions

In this study, we used a continuous motion judgment task to assess the relative contribution of two different report methods and three different versions of the RDK. Our data revealed that a report in a pure motor framework is strongly biased (presumably by motor ergonomy) and adds substantial motor variability to behavior responses compared with a pure perceptual report method that was independent of directional motor movements. By means of a perceptual report method in combination with a modeling approach, we were able to determine differences between the three most commonly applied RDKs that suggest behavior performance in TM to differ more substantially from BM and WM. Independent of report and stimulus modality, we detected a systematic misperception where subjects responded in the opposite of the presented direction in a substantial number of trials. Taken together, our findings suggest that research on continuous report tasks should consider the impact of perceptual and motor variability inherent in their experimental design when interpreting behavioral or neural data.

## Appendix

### vMMM

Based on previous research (Zhang & Luck, 2008; Bays et al., 2009; Ma et al., 2014; Bae and Luck, 2019), we hypothesized that the distribution of response deviations Δθ consisted of three components: i) trials in which the direction of motion was identified with a specific precision, ii) ROODs with a specific precision, and iii) trials in which subjects were guessing the true direction (cf. Figure 8). This motivates a three-component mixture model with the following event types:

**Table:**

	
*A_i_*:	the response in trial *i* is a detection;
*B_i_*:	the response in trial *i* is a ROOD;
*C_i_*:	the response in trial *i* is guessing.

Detection responses are assumed to follow a vM distribution with mean 0° plus μ (where μ is the bias term) and precision κ_1_:

(4) $$\begin{array}{c} p\left(\theta_{i}|A_{i},\mu ,\kappa_{1}\right)=\mathrm{vM}\left(\theta_{i};0^{\circ }+\mu ,\kappa_{1}\right).\; \end{array}$$

ROOD responses are assumed to follow a von Mises (vM) distribution with mean 180° plus bias μ and precision κ_2_:

(5) $$\begin{array}{c} p\left(\theta_{i}|B_{i},\mu ,\kappa_{2}\right)=\mathrm{vM}\left(\theta_{i};180^{\circ }+\mu ,\kappa_{2}\right).\; \end{array}$$

Finally, guessing responses are assumed to follow a continuous uniform (U) distribution between 0° and 360°:

(6) $$\begin{array}{c} p\left(\theta_{i}|C_{i}\right)=U\left(\theta_{i};0^{\circ },360^{\circ }\right).\; \end{array}$$

The individual event probabilities, equivalent to the mixture coefficients, are given by:

(7) $$\begin{array}{ccc} & & p\left(A_{i}\right)=r_{1},\;p\left(B_{i}\right)=r_{2},\\ & & p\left(C_{i}\right)=r_{3}=1-r_{1}-r_{2}.\; \end{array}$$

Using the law of marginal probability, we obtain the probability of observing, in an individual trial, the response deviation θ_*i*_, given model parameters *r*_1_, *r*_2_, κ_1_, and κ_2_:

(8) $$\begin{array}{ccc} \;\;\;\;\;\;\;\;\;\;\;\;p(\theta_{i}|r_{1},r_{2},\mu ,\;\kappa_{1},\;\kappa_{2}) & \;= & p\left(\theta_{i}|A_{i},\mu ,\kappa_{1}\right)\;p\left(A_{i}\right)\\ & & \;+p\left(\theta_{i}|B_{i},\mu ,\kappa_{2}\right)\;p\left(B_{i}\right)\\ & & \;+p\left(\theta_{i}|C_{i}\right)\;p\left(C_{i}\right), \end{array}$$

(9) $$\begin{array}{ccc} \;\;\;\;\;\;\;\;\;\;\;\;p\left(\theta_{i}|r_{1},r_{2},\mu ,\;\kappa_{1},\;\kappa_{2}\right) & \;= & r_{1}\cdot \mathrm{vM}\left(\theta_{i};0^{\circ }+\mu ,\kappa_{1}\right)\\ & & \;+r_{2}\cdot \mathrm{vM}\left(\theta_{i};180^{\circ }+\mu ,\kappa_{2}\right)\\ & & \;+r_{3}\cdot U\left(\theta_{i};0^{\circ },360^{\circ }\right). \end{array}$$

With the probability density function of the vM distribution

(10) $$p\left(x|\mu ,\kappa \right)=\frac{\exp\left[\kappa \;\cos\left(x-\mu \right)\right]}{2\pi \;I_{0}\left(\kappa \right)},$$

where *I_p_*(*x*) is the modified Bessel function of order *p*, and the density of the continuous uniform distribution is

(11) $$p\left(x|a,b\right)=\frac{1}{b-a}\;,$$

the probability of an individual observation evaluates to

(12) $$\begin{array}{ccc} \; & & p\left(\theta_{i}|r_{1},r_{2},\mu ,\;\kappa_{1},\;\kappa_{2}\right)\\ \; & & =r_{1}\cdot \frac{\exp\left[\kappa_{1}\;\cos\left(\theta_{i}-\mu \right)\right]}{2\pi \;I_{0}\left(\kappa_{1}\right)}\\ \; & & \;+r_{2}\cdot \frac{\exp\left[\kappa_{2}\;\cos\left(\theta_{i}-\left(180^{\circ }+\mu \right)\right)\right]}{2\pi \;I_{0}\left(\kappa_{2}\right)}+r_{3}\cdot \frac{1}{2\pi }\;,\;\;\;\;\; \end{array}$$

which is the core equation of the vMMM. Note that this differs from earlier proposals (Bae & Luck, 2019) by the fact that it assumes different precisions for correct responses (κ_1_) and 180° errors (κ_2_) and that it assumes a nonzero response bias (μ). The vMMM thus comprises five free model parameters, detection and ROOD frequencies *r*_1_ and *r*_2_, and detection and ROOD precisions κ_1_ and κ_2_, as well as the response bias μ. Note that the guessing frequency *r*_3_ is not a free parameter, because *r*_3_ = 1 − *r*_1_ − *r*_2_.

Assuming independence between trials entails that individual likelihoods factorize, so that we obtain the likelihood function for the entire data

(13) $$\begin{array}{ccc} & & p(y|r_{1},r_{2},\mu ,\;\kappa_{1},\;\kappa_{2})\\ & & =\prod_{i=1}^{n}p\left(\theta_{i}|r_{1},r_{2},\mu ,\;\kappa_{1},\;\kappa_{2}\right),\; \end{array}$$

and the corresponding log-likelihood function

(14) $$\begin{array}{ccc} & & \log p(y|r_{1},r_{2},\mu ,\;\kappa_{1},\;\kappa_{2})\\ & & =\sum_{i=1}^{n}\log p\left(\theta_{i}|r_{1},r_{2},\mu ,\;\kappa_{1},\;\kappa_{2}\right).\; \end{array}$$

### Model space

Based on the general vMMM formulation, one can force some of the mixture coefficients to zero, giving rise to reduced versions of the vMMM:

**Table:**

	
*m*_00_:	*r*_1_ = *r*_2_ = 0;
*m*_01_:	*r*_1_ = 0;
*m*_10_:	*r*_2_ = 0;
*m*_11_:	*r*_1_,*r*_2_ ≠ 0.

Here, *m*_00_ corresponds to the assumption that effectively all the responses of a subject are guessing (only guessing); *m*_01_ additionally includes the possibility of ROODs (ROOD and guessing); *m*_10_ additionally includes the possibility of detections (detection and guessing); and *m*_11_ corresponds with the full model assuming all three processes (detection, ROOD, and guessing). Note that the guessing component cannot be removed (*r*_3_ = 0) from *m*_00_, because it represents noise-level performance. Whereas *m*_01_ is an a priori entirely implausible model (there should not be ROOD when there is no detection), *m*_00_ should perform well in very low coherence cases (e.g., 0%); *m*_10_ should perform well in very high coherence (e.g., 100%); and *m*_11_ should perform well on medium coherence levels (e.g., 50%).

### Model estimation

Classical model estimation is performed via maximum likelihood estimation:

(15) $$\begin{array}{ccc} \; & & \left({\hat{r}}_{1},{\hat{r}}_{2},\hat{\mu },{\hat{\kappa }}_{1},{\hat{\kappa }}_{2}\right)\\ \; & & =\arg\;\mathrm{ma}x_{\left(r_{1},r_{2},\mu ,\;\kappa_{1},\;\kappa_{2}\right)}\;p\left(y|r_{1},r_{2},\mu ,\;\kappa_{1},\;\kappa_{2}\right).\; \end{array}$$

This gives rise to the maximum log-likelihood (MLL) as a measure of model fit:

(16) $$\mathrm{MLL}=\log p(y|{\hat{r}}_{1},{\hat{r}}_{2},\hat{\mu },{\hat{\kappa }}_{1},{\hat{\kappa }}_{2}).$$

In this work, the maximum likelihood estimation was implemented via a refined grid search algorithm.

### Model comparison

Classical model comparison rests on information criteria such as Akaike's information criterion (AIC; Akaike, 1974; Burnham & Anderson, 2002; Wagenmakers & Farrell, 2004), which can be calculated from the MLL

(17) $$\begin{array}{c} \mathrm{AIC}\left(m_{i}\right)=-2\;\mathrm{MLL}\left(m_{i}\right)+2\;k,\; \end{array}$$

where *i* indexes the model and *k* is the number of free model parameters (μ: *m*_00_: 0; *m*_01_: 3; *m*_10_: 3; *m*_11_: 5). Raw AIC values have no absolute meaning, but can be recalculated into more intuitive Akaike weights (AIC_w_) in the following way:

(18) $$\begin{array}{c} \Delta \mathrm{AIC}\left(m_{i}\right)=\mathrm{AIC}\left(m_{i}\right)-\min_{m}\mathrm{AIC}\left(m\right),\; \end{array}$$

(19) $$\begin{array}{c} \mathrm{AI}C_{w}\left(m_{i}\right)=\frac{\exp\left[-\frac{1}{2}\Delta \mathrm{AIC}\left(m_{i}\right)\right]}{\sum_{m}\exp\left[-\frac{1}{2}\Delta \mathrm{AIC}\left(m\right)\right]}.\; \end{array}$$

Akaike weights represent a normalization to model space and, therefore, always require a set of candidate models. They always fall between 0 and 1 and thus have an interpretation that is similar to the one of posterior model probabilities (as discussed elsewhere in this article).

### Model averaging

The components underlying the distribution of response deviations might be dependent on the coherence level and RDK type. To account for this variability, model averaging was applied (Soch, Meyer, Haynes, & Allefeld, 2017). This procedure allowed us to weight the influence of each component for each coherence level and RDK, to obtain the optimal parameter estimates for each condition. This analysis was performed separately for each subject to allow for statistical comparisons of the estimated parameters.

In quasi-Bayesian model averaging (MA), averaged estimates are given by a weighted sum of model-wise parameter estimates where the weights are the posterior probabilities of the models used to estimate them:

(20) $${\hat{\phi }}_{\mathrm{MA}}=\sum_{i=1}^{M}{\hat{\phi }}_{i}\cdot p\left(m_{i}|y\right).$$

In this work, MA was implemented using ML estimates for the model-wise parameter estimates and Akaike weights as posterior model probabilities:

(21) $$\hat{r}=\sum_{i=1}^{M}{\hat{r}}^{\left(i\right)}\cdot \mathrm{AI}C_{w}\left(m_{i}\right),$$

(22) $$\hat{\mu }=\sum_{i=1}^{M}{\hat{\mu }}^{\left(i\right)}\cdot \mathrm{AI}C_{w}\left(m_{i}\right),$$

(23) $$\hat{\kappa }=\sum_{i=1}^{M}{\hat{\kappa }}^{\left(i\right)}\cdot \mathrm{AI}C_{w}\left(m_{i}\right).$$

In this way, modeling uncertainty is decreased in the estimation process, such that model-averaged parameter estimates are likely to be more precise than estimates obtained using the same model for all coherence levels. For example, in very low coherence cases (e.g., 0%), parameter estimates will be dominated by those from *m*_00_. Conversely, in very high coherence situations (e.g. 100%), parameter estimates should be dominated by *m*_10_ or *m*_11_, depending on whether there is evidence for ROODs (*m*_11_) or not (*m*_10_).
